# Motivations and barriers to uptake and use of female-initiated, biomedical HIV prevention products in sub-Saharan Africa: an adapted meta-ethnography

**DOI:** 10.1186/s12889-017-4959-3

**Published:** 2017-12-19

**Authors:** Robyn Eakle, Adam Bourne, Caitlin Jarrett, Jonathan Stadler, Heidi Larson

**Affiliations:** 1 Wits Reproductive Health & HIV Institute, University of the Witwatersrand, Hillbrow Health Precinct, 22 Esselen Street, Hillbrow, Johannesburg, 2001 South Africa; 2 Department of Social and Environmental Health Research, Sigma Research, London School of Hygiene and Tropical Medicine, London, UK; 3 Australian Research Centre in Sex, Health & Society, La Trobe University, Melbourne, Australia; 4 Department of Infectious Disease Epidemiology, London School of Hygiene and Tropical Medicine, London, UK; 5 Swiss Tropical and Public Health Institute (Swiss TPH), Basel, Switzerland; 6 University of Basel, Basel, Switzerland; 7 Department of Anthropology and Development Studies, University of Johannesburg, Johannesburg, South Africa

**Keywords:** HIV prevention, Biomedical prevention products, Pre-exposure prophylaxis (PrEP), Women, Qualitative research

## Abstract

**Background:**

Women bear a disproportionate burden of HIV throughout the world prompting extensive research into HIV prevention products for women which has met with varied success. With an aim of informing future policy and programming, this review examines the barriers and motivations to the uptake and use of female initiated products in sub-Saharan countries.

**Methods:**

We conducted a systematic review as an adapted meta-ethnography of qualitative data focused on actual use of products. After deduplication, 10,581 and 3861 papers in the first and second round respectively were screened. Following the PRISMA guidance, 22 papers were selected and synthesized using Malpass’s definitions of first, second, and third order constructs. First order constructs, consisting of participant data published in the selected papers, were extracted and categorised by second and third order constructs for analysis. A weight of evidence review was conducted to compare and assess quality across the papers.

**Results:**

The 22 papers selected span 11 studies in 13 countries. We derived 23 s order constructs that were translated into seven overarching third order constructs: Sexual Satisfaction, Trust, Empowerment and Control, Personal Well-being, Product use in the social-cultural environment, Practical Considerations, Risk Reduction, and Perceptions of Efficacy. Relationships and trust were seen to be as or more important for product use as efficacy. These constructs reveal an inherent inter-relationality where decision making around HIV prevention uptake and use cannot be binary or mono-faceted, but rather conducted on multiple levels. We developed a framework illustrating the central and proximal natures of constructs as they relate to the decision-making process surrounding the use of prevention products.

**Conclusions:**

Health systems, structural, and individual level HIV prevention interventions for women should adopt a holistic approach. Interventions should attend to the ways in which HIV prevention products can serve to reduce the likelihood of HIV transmission, as well as help to protect partnerships, enhance sexual pleasure, and take into account woman’s roles in the social environment. Stigma, as well as sexuality, is likely to continue to influence product uptake and use and should be prominently taken into account in large-scale interventions.

**Trial registration:**

Not applicable.

## Background

Women bear a disproportionate burden of HIV infection across the world, and in particular in sub-Saharan Africa [1]. Until recently, the only readily available HIV prevention options for women have been male condoms. Female condoms were at one time a promising new option, however lack of support from international agencies and funders translated into challenges in delivery and access [2–4]. This meant that male condoms have remained the dominant form of HIV prevention for decades. Additionally, post-exposure prophylaxis (PEP), while proven to be efficacious in preventing HIV acquisition [5, 6], has generally only been available for health workers and rape victims [7, 8].

Advances in HIV prevention research have yielded a new approach: pre-exposure prophylaxis (PrEP). PrEP is the use of antiretroviral drugs taken orally by people who do not have HIV to prevent acquisition of the virus. A recent systematic review of oral PrEP including 18 studies found that “PrEP use with greater than 70% adherence demonstrated the highest PrEP effectiveness (RR = 0.30, 95% CI: 0.21-0.45, *p* < 0.001) compared to placebo”, confirming that oral PrEP will prevent HIV with high rates of efficacy when taken consistently [9]. This review did not include one study completed with people who inject drugs [10], which found a moderate but significant level of efficacy, nor did it include two microbicide gel^1^ studies (CAPRISA004 and FACTS001), the results of which together did not prove product efficacy [11, 12]. The non-significant levels of efficacy in the two microbicide trials, as well as the similar results of the VOICE (comparing oral PrEP and microbicide gel) and FEMPrEP (oral PrEP only) trials were either partially or largely due to poor adherence [13, 14], opening up questions around the ability to take oral PrEP effectively.

Qualitative research conducted as part of these clinical trials has explored reasons for poor adherence. Reasons range from apathy towards the research itself, dislike of product side effects, lack of privacy in which to use the products, low risk perception, and access to better healthcare offered in studies as primary motivation for study participation [15, 16]. The insights arising from these studies, conducted primarily in sub-Saharan Africa and India, combined with lessons learned from past research of other HIV prevention products will provide the field with further understanding of why and how women take up and use HIV prevention products, which can inform better implementation.

This is a systematic literature review conducted in the form of a meta-ethnography to synthesize qualitative findings from research on the practical use of HIV prevention products among populations of women across sub-Saharan Africa. Broadly, our aim is to inform future policy and programming for HIV prevention products for women going forward, by drawing on the wealth of information already published from the research conducted in sub-Saharan Africa. As such, the primary objective of this review is to identify and understand the motivations and barriers affecting uptake and use of female-initiated, primary biomedical HIV prevention products for women in sub-Saharan Africa.

## Methods

We conducted a systematic review using a meta-ethnographic approach following the principles set out by Noblit and Hare [17]. This approach allows for a sophisticated and robust manner of synthesis as compared to a typical literature review of qualitative data, and focuses on interpretation through analysis of constructs rather than summarization of themes [18]. For this review, we conducted an adapted meta-ethnography as defined more recently by several researchers [19–21], which allows for qualitative data collected through a variety of methods, such as interviews and focus groups, as well as ethnographies, to be combined and interpreted. Qualitative data are best placed to answer the questions around how and why products are utilised effectively, rather than measuring only their uptake or adherence. Throughout this process, we also employed the guidance set forth by the Preferred Reporting Items for Systematic Reviews and Meta-Analyses (PRISMA) Statement and the Centre for Reviews and Dissemination (CRD) [22, 23]. The protocol for this review has been published and the methods described in more detail [24].

This review was not registered on PROSPERO since reviews of qualitative evidence are not currently included on the PROSPERO database. Additionally, the original protocol articulated “female-initiated HIV prevention technologies” in the title, however we chose to change technologies to products in the final paper.

### Search strategy and inclusion criteria

We searched seven databases: Africa-wide Info, CINAHL, Embase, Global Health, Medline, Psychinfo, and Web of Science. The search strategy comprised four primary concepts: HIV prevention; uptake and use; qualitative research; and sub-Saharan Africa, which were first confirmed through iterative pilot searching in Medline, and then adapted for the other databases. The first set of searches was conducted in July of 2013, and then again in July of 2015.

Papers were included in the review if they met the following criteria: women aged 18 and above; data focused on female-initiated products (oral PrEP, microbicide gel, PEP, female condom, vaginal ring, and diaphragms); included narrative on motivations and/or barriers to uptake and use of products; qualitative research; located in sub-Saharan Africa; and, research conducted from 2003 or later. Note that female-initiated product refers to any HIV prevention product that can be initiated and used exclusively by women without requiring the involvement or permission of a partner.

Actual experience of product use was central to this review, rather than hypothetical acceptability studies (e.g. where study participants did not actually have access to products). Since few studies have been published in ‘real-world’ programme settings, we also included data from across research settings, both randomized control trial and implementation. While incentives to participate in research could be quite different to clinic attendance, in this review we hypothesized that experiences of actual product use should be similar regardless of initial motivation. Additionally, we have included women’s perspectives on use of the diaphragm and vaginal microbicide gels, despite the limited efficacy of these products to prevent HIV. At the time of those studies, efficacy was unknown, and importantly, women’s experiences and perceptions of use extend beyond efficacy. The interest of this review is to examine the elements that would make a product feasible and relevant for a woman to use it, and what those salient elements are across products.

We did not limit our search by language, and we allowed for grey literature to be included, however none was identified through our searches or through consultation with relevant subject matter experts. Studies were excluded if they focused only on hypothetical use of products or represented perspectives from the male point of view, HIV-positive women only, or secondary prevention products (e.g. Prevention of Mother to Child Transmission).

### Screening and selection process

**Fig. 1:**
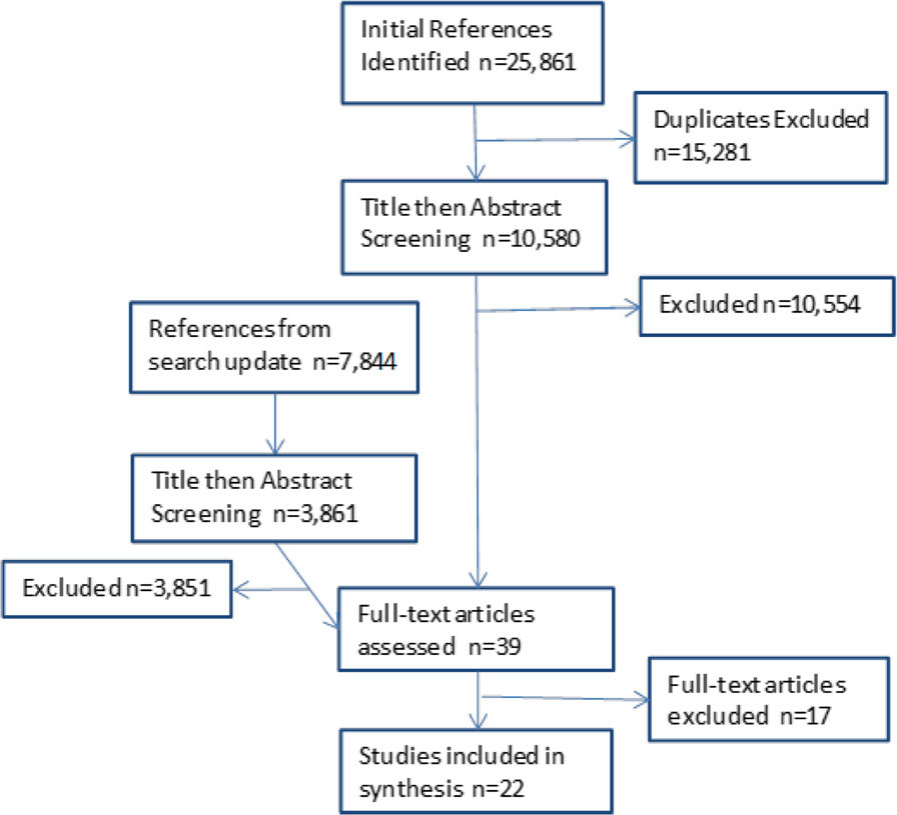
PRISMA chart of review process

All 25,861 references identified through searching were uploaded into the reference software manager Mendeley. After de-duplication, two reviewers screened all 10,580 papers by title and abstract according to the inclusion and exclusion criteria. Any discrepancies between reviewers were discussed and mediated by a third researcher. The papers were identified first by title and then by abstract, and were then reviewed in duplicate by three reviewers. There were several cases of papers published from the same study, however we determined no overlap of data therefore these papers were all included. The 39 papers identified for possibility for inclusion were discussed by the three primary reviewers during which some were eliminated mainly due to inability to isolate female-centred data from male, or due to the research being conducted before 2003. Finally, 22 papers were selected for analysis. This process is illustrated in Fig. 1.

### Analysis and synthesis

Data were extracted from the papers by two reviewers, then sorted by themes and incorporated into a construct worksheet. To generate the concepts for our constructs, we employed Malpass’s definition of the first, second, and third order constructs [25] used previously by authors of other similar reviews [19, 20]. First order constructs consist of participant data published in the selected papers, and were extracted and categorised by second and third order constructs for analysis. Second order constructs consist of author perspectives of their manuscript data extracted from the papers, and third order constructs are thematic categories developed through our analysis. In this regard, our construct worksheet comprised three meta-ethnographic layers: the perspective of the participants, the perspective of the paper authors, and the perspectives of the researchers conducting this review. We then used the process of concept translation, as described by Musheke et al. [20], to arrive at our synthesized third order constructs.

### Weight of evidence review

We employed a weight of evidence (WoE) review to assess the relative strength of the papers included in the review. A WoE review, as defined by Gough et al. [26], is a process by which standard elements of research are identified in each paper included in the review, and then assessed in comparison with one another to judge the overall strength or quality of the papers [26]. The results of our WoE are listed in Table 1. This process uses a systematic approach similar to the GRADE process used by the World Health Organization (WHO) in assessing the quality of quantitative studies included in systematic reviews in support of guidelines. Each paper was assessed in terms of relevance (how directly the paper answered the aim of the review), appropriateness of study design, and soundness (which equates to the inclusion criteria for this review).

**Table 1:** List of papers and Weight of Evidence Review

Author	Title	Publication Year	Product	Data Type	Study Context	Location	Population	N	Study Dates	Theory	Soundness	Appropriateness of study design	Relevance	Overall Rating
Abrahams, Naeemah & Jewkes, Rachel	Barriers to post exposure prophylaxis [PEP] completion after rape: a South African qualitative study	2010	PEP	IDIs	Stand-alone qualitative	South Africa	Victims of sexual assault	29	2005-2006	Not specified	Medium-High: no theoretical approach articulated for study	High: standalone qualitative research	High: though an outlier, specifically discusses barriers to PEP use	High
Behets, Frieda M T F; Van Damme, Kathleen; Turner, Abigail Norris; Rabenja, Ny Lovaniaina; Ravelomanana, Noro L R; Raharinivo, Mbolatiana S M; Zeller, Kimberly A; Rennie, Stuart M & Swezey, Teri A	Evidence-based planning of a randomized controlled trial on diaphragm use for prevention of sexually transmitted infections	2008	Diaphragm	FGDs	Formative, qualitative	Madagascar	Female sex workers	266	2004	Not specified	Medium-High: no theoretical approach articulated for study	Medium-High: qualitative research within a formative research activity, mixed methods	Medium-High: qualitative research focusing on acceptability, data on motivations and barriers come through	Medium-High
Gafos, Mitzy; Mzimela, Misiwe; Sukazi, Sizakele; Pool, Robert; Montgomery, Catherine & Elford, Jonathan	Intravaginal insertion in KwaZulu-Natal: sexual practices and preferences in the context of microbicide gel use	2010	Pro2000 gel	IDIs and FGDs	MDP 301 Phase III RCT	South Africa	Sexually active adultwomen/trial participants	136	March 2006 - August 2008	Not specified	Medium-High: no theoretical approach articulated for study	Medium-High: qualitative research within a larger trial setting	Medium-High: qualitative research on vaginal practices	Medium-High
Greene, Elizabeth; Batona, Georges; Hallad, Jyoti; Johnson, Sethulakshmi; Neema, Stella & Tolley, Elizabeth E	Acceptability and adherence of a candidate microbicide gel among high-risk women in Africa and India	2010	Celulose Sulfate	IDIs	Phase III RCT	Uganda, Benin	High risk women/trial participants	30	Feb-Aug 2007	A variation of the socio-ecological model (Mcleroy et al. 1988)	High - all details included	Medium-High: qualitative research within a larger trial setting	High - specifically evaluates barriers to use of gel among users	High
Guest, Greg; Johnson, Laura; Burke, Holly; Rain-Taljaard, Reathe; Severy, Lawrence; von Mollendorf, Claire & Van Damme, Lut	Changes in sexual behavior during a safety and feasibility trial of a microbicide/diaphragm combination: an integrated qualitative and quantitative analysis	2008	ACIDFORM Gel and Diaphragm	IDIs and FGDs	Safety and Feasibility study	South Africa	Sexually active adult women	120	April 2004 - Nov 2005	Data analysis conducted within a positivist framework (Bernard & Ryan, 1998), no specific theory for study	Medium-High: no theoretical approach articulated for study	Medium-High: qualitative research within a larger trialsetting	Medium: paper is focused on changes in sexual behaviour and not motivators/barriers to use, although these come out in the data	Medium-High
Guest, G; Shattuck, D; Johnson, L; Akumatey, B; Clarke, E E K; Chen, P & MacQueen, K M	Acceptability of PrEP for HIV prevention among women at high risk for HIV	2010	Oral TDF PrEP	IDIs	Phase III RCT	Nigeria, Cameroon, Ghana	Sexually active adult women/trial participants	24	June 2004-March 2006	Not specified	Medium-High: no theoretical approach articulated for study	Medium-High: qualitative research within a larger trial setting	Medium-High: qualitative research focusing on acceptability, data on motivations and barriers come through	Medium-High
Kacanek, Deborah; Dennis, Amanda; Sahin-Hodoglugil, Nuriye; Montgomery, Elizabeth T; Morar, Neetha; Mtetwa, Sibongile; Nkala, Busi; Phillip, Jessica; Watadzaushe, Connie & Van, der Straten	A qualitative study of obstacles to diaphragm and condom use in an HIV prevention trial in sub-Saharan Africa	2012	Diaphragm	FGDs	MIRA Trial phase III RCT	South Africa and Zimbabwe	Sexually active adult women/trial participants	206	Aug 2006 - Jan 2007	Modified grounded theory (Glaser & Strauss, 1967) for analysis, no specific theory for study	Medium-High: no theoretical approach articulated for study	Medium-High: qualitative research within a larger trial setting	Medium-High: data focused on ability to use condoms with diaphragm, though barriers to use of diaphragm came through	Medium-High
Mathenjwa, T & Maharaj, P	Female condoms give women greater control’: A qualitative assessment of the experiences of commercial sex workers in Swaziland	2012	Female condom	IDIs and FGDs	Stand-alone qualitative	Swaziland	Female sex workers	25	Jan - May 2010	Not specified	Medium-High: no theoretical approach articulated for study	High: standalone qualitative research	High: specifically looks at experiences, motivations and barriers to use of female condom	High
Montgomery, C M; Lees, S; Stadler, J; Morar, N S; Ssali, A; Mwanza, B; Mntambo, M; Phillip, J; Watts, C & Pool, R	The role of partnership dynamics in determining the acceptability of condoms and microbicides	2008	Pro2000 gel	IDIs	Component of pilot study for MDP 301 phase III randomized trial	South Africa, Tanzania, Uganda and Zambia	general population women in couples	45	Not specified	none specified (though used relationship based questions and anthropological approaches)	Medium: study dates not specified, though can assess date of data collection knowing this was connected with larger MDP301 study; no theoretical approach articulated for the study	Medium-High: qualitative research within a pilot for a larger trial	High: specifically evaluates experiences of gel use	Medium-High
Montgomery, Catherine M; Gafos, Mitzy; Lees, Shelley; Morar, Neetha S; Mweemba, Oliver; Ssali, Agnes; Stadler, Jonathan & Pool, Robert	Re-framing microbicide acceptability: findings from the MDP301 trial	2010	Pro2000 gel	Semi-structured, serial IDIs	Component of MDP 301 phase III randomized trial	South Africa, Zambia, Uganda and Tanzania	Sexually active adult women/trial participants	464	The trial started in October 2005 and completed followup in August 2009	emic approach to acceptability	High: all details included	Medium-High: qualitative research within a larger trial setting	High: specific to women’s experiences of gel, and their interpretations of use	High
van der Straten, A, Montgomery, Elizabeth T; Straten, A; Cheng, H; Wegner, L; Masenga, G; Mollendorf, C; Bekker, L; Ganesh, S; Young, K; Romano, J; Nel, A; Woodsong, C; & von Mollendorf, C	High Acceptability of a Vaginal Ring Intended as a Microbicide Delivery Method for HIV Prevention in African Women	2012	Placebo vaginal ring	FGDs	randomized safety and acceptability study (mixed methods)	South Africa and Tanzania	Sexually active adult women/trial participants	48	April 2007 to March 2010	Not specified	Medium-High: no theoretical approach articulated for study	High: qualitativeresearch within a pilot/acceptability study	High: specifically aimed at understanding possible motivations and barriers to use of the ring	High
Montgomery, Elizabeth T; Chidanyika, Agnes; Chipato, Tsungai; Van, der Straten; Montgomery T, Elizabeth & Ariane	Sharing the trousers: gender roles and relationships in an HIV-prevention trial in Zimbabwe	2012	MIRA diaphragm and replens lubricant	FGDs and IDIs	MIRA Male Involvement Study (ancillary to MIRA trial)	Zimbabwe	Sexually active adult women/trial participants	85	August 2006 to June 2007	Not specified	Medium-High: no theoretical approach articulated for study	Medium-High: qualitative research within a larger trial setting	Medium: specifically looks at gender roles around decision making in the house and around sex, but experiences with diaphragm come through	Medium-High
Okal, Jerry; Stadler, Jonathan; Ombidi, Wilkister; Jao, Irene; Luchters, Stanley; Temmerman, Marleen & Chersich, Matthew F	Secrecy, disclosure and accidental discovery: perspectives of diaphragm users in Mombasa, Kenya	2008	Diaphragm	IDIs and FGDs	prospective study investigating diaphragm continuation rates	Kenya	Sexually active adult women	39	January 2004 - July 2005	None specified	Medium-High: no theoretical approach articulated for study	High: standalone qualitative research	High: specifically aimed at understanding possible motivations and barriers to use of the diaphragm	High
Sahin-Hodoglugil, Nuriye; Montgomery, Elizabeth; Kacanek, Deborah; Morar, Neetha; Mtetwa, Sibongile; Nkala, Busisiwe; Philip, Jessica; Ramjee, Gita; Cheng, Helen; Ariane; SahinHodoglugil, N N; Straten, A & Team, The Mira	User experiences and acceptability attributes of the diaphragm and lubricant gel in an HIV prevention trial in southern Africa	2011	MIRA diaphragm and replens lubricant	FGDs	MIRA Trial phase III RCT	Zimbabwe and South Africa	Sexually active adult women/trial participants	105	August 2006 to January 2007	None specified	Medium-High: no theoretical approach articulated for study	Medium-High: qualitative research within a larger trial setting	High: specifically evaluates experiences of diaphragm and gel use	Medium-High
Stadler, Jonathan & Saethre, Eirik	Blockage and flow: intimate experiences of condoms and microbicides in a South African clinical trial	2011	Pro2000 gel	IDIs, FGDs, and participant observation	Qualitative research conducted during MDP301 phase III efficacy trial	South Africa	Sexually active adult women/trial participants	179 women in 401 IDIs, 42 FGDs	Trial was completed in August 2008 and follow upcompleted in August 2009	Not specified	Medium-High: no theoretical approach articulated for study	Medium-High: qualitative research within a larger trial setting	Medium-High: examined women’s interpretation and meanings of condom and gel use; leads to motivations and barriers but not explictily examining	Medium-High
van der Straten A, Stadler J, Montgomery E, Hartmann M, Magazi B, Mathebula F, Schwartz K, Laborde N, Soto-Torres L.	Women’s Experiences with Oral and Vaginal Pre-Exposure Prophylaxis: The VOICE-C Qualitative Study in Johannesburg, South Africa.	2014	TDF gel and Oral TDF and Truvada	IDIs, serial ethnographic interviews, FGDs, observations	Qualitative sub-study in VOICE phase III randomized clinical trial	South Africa	Sexually active adult women/trial participants	102	July 2010 and August 2012	social-ecological model	High: all details included	High: qualitative sub-study for larger trial	High: specifically examines user experiences of gel and pill use	High
Gafos, Mitzy; Pool, Robert; Mzimela, Misiwe Adelaide; Ndlovu, Hlengiwe Beauty; McCormack, Sheena; Elford, Jonathan & Team, M D P	The implications of post-coital intravaginal cleansing for the introduction of vaginal microbicides in South Africa	2014	Pro2000 gel	serial ethnographic interviews	Qualitative research conducted during MDP301 phase III efficacy trial	South Africa	Sexually active adult women/trial participants	84	March 2006 to August 2008 with follow-up visits continuing until August 2009	Not specified	Medium-High: no theoretical approach articulated for study	Medium-High: qualitative research within a larger trial setting	Medium - explores vaginal hygiene practices within context of gel use	Medium-High
Lees, S	Emergent HIV technology: urban Tanzanian women’s narratives of medical research, microbicides and sexuality	2015	Pro2000 gel	IDIs and observations	Qualitative research conducted during MDP301 phase III efficacy trial	Tanzania	Sexually active adult women/trial participants	99	November 2005 to August 2009	None specified (though used anthropological approach)	Medium-High: no theoretical approach articulated for study	Medium-High: qualitative research within a larger trial setting	Medium-High: specifically explores motivations for participating in research, but also includes experiences and interpretations of gel use	Medium-High
Magazi, Busisiwe; Stadler, Jonathan; Delany-Moretlwe, Sinead; Montgomery, Elizabeth; Mathebula, Florence; Hartmann, Miriam & van der Straten, Ariane	Influences on visit retention in clinical trials: insights from qualitative research during the VOICE trial in Johannesburg, South Africa	2014	TDF gel and Oral TDF and Truvada	IDIs and FGDs	Qualitative sub-study in VOICE phase III randomized clinical trial	South Africa	Sexually active adult women/trial participants	102	July 2010 to August 2012	social-ecological model	High: all details included	High: qualitative sub-study for larger trial	High: specifically examines user experiences of gel and pill use	High
Montgomery, Elizabeth T; van der Straten, Ariane; Stadler, Jonathan; Hartmann, Miriam; Magazi, Busisiwe; Mathebula, Florence; Laborde, Nicole & Soto-Torres, Lydia	Male partner influence on women’s hiv prevention trial participation and use of pre-exposure prophylaxis: The importance of understanding	2015	TDF gel and Oral TDF and Truvada	IDIs and FGDs	Qualitative sub-study in VOICE phase III randomized clinical trial	South Africa	Sexually active adult women/trial participants	102	July 2010 to August 2012	social-ecological model	High: all details included	High: qualitative sub-study for larger trial	Medium-High: looked more at partnership dynamics than experiences of product use, but influences of dynamics on use is explored	High
Stadler, J; Delany-Moretlwe, S; Palanee, T & Rees, H	Hidden harms: women’s narratives of intimate partner violence in a microbicide trial, South Africa	2014	Pro2000 gel	serial IDIs	Qualitative research conducted during MDP301 phase III efficacy trial	South Africa	Sexually active adult women/trial participants	401 IDIs with 150 women	Not actually specified except “up to 2010” (see other MDP papers)	None specified	Medium: study dates not specified, though can assess date of data collection knowing this was connected with larger MDP301 study; no theoretical approach articulated for the study	Medium-High: qualitative research within a larger trial setting	Medium-High: looked more at partnership dynamics than experiences of product use, but influences of dynamics on use is explored	Medium-High
Van Der Straten, A; Stadler, J; Luecke, E; Laborde, N; Hartmann, M & Montgomery, E T	Perspectives on use of oral and vaginal antiretrovirals for HIV prevention: The VOICE-C qualitative study in Johannesburg, South Africa	2014	TDF gel and Oral TDF and Truvada	IDIs and FGDs	Qualitative sub-study in VOICE phase III randomized clinical trial	South Africa	Sexually active adult women/trial participants	102	July 2010 to August 2012	social-ecological model	High: all details included	High: qualitative sub-study for larger trial	High: specifically examines user experiences of gel and pill use, and in particular how meanings of ARVs for prevention can become conflated with treatment and being HIV +	High

## Results

**Fig. 2:**
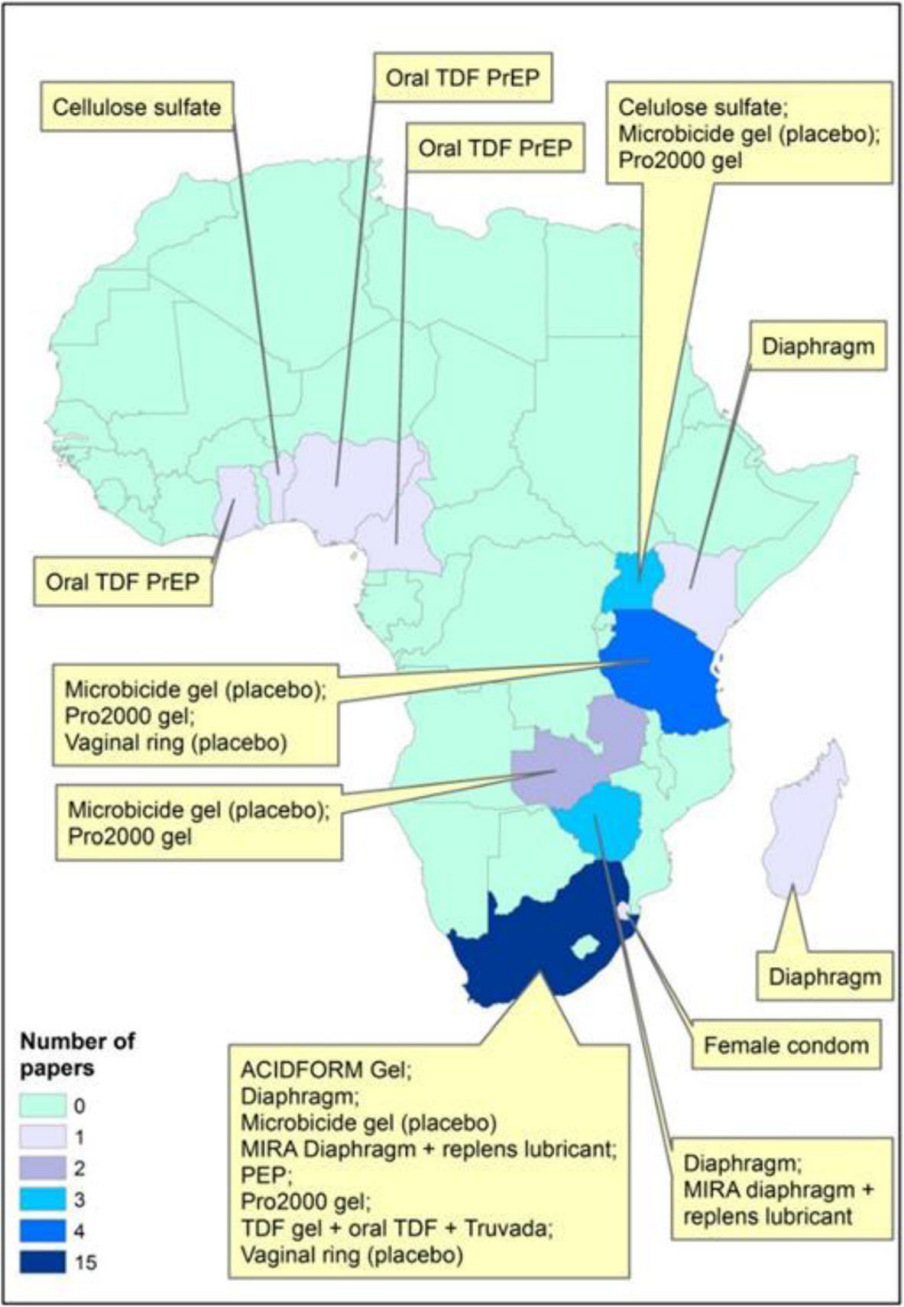
Map indicating HIV prevention products

The 22 papers included in the review represent 11 studies (including ancillary research as part of larger studies) across 13 countries in sub-Saharan Africa, as shown in Fig. 2. Since the review covers studies conducted between 2003 and 2015, more papers describe experiences using products such as diaphragms and microbicide gels, as compared to more recent products such as oral PrEP for which few papers have yet been published.

**Fig. 3:**
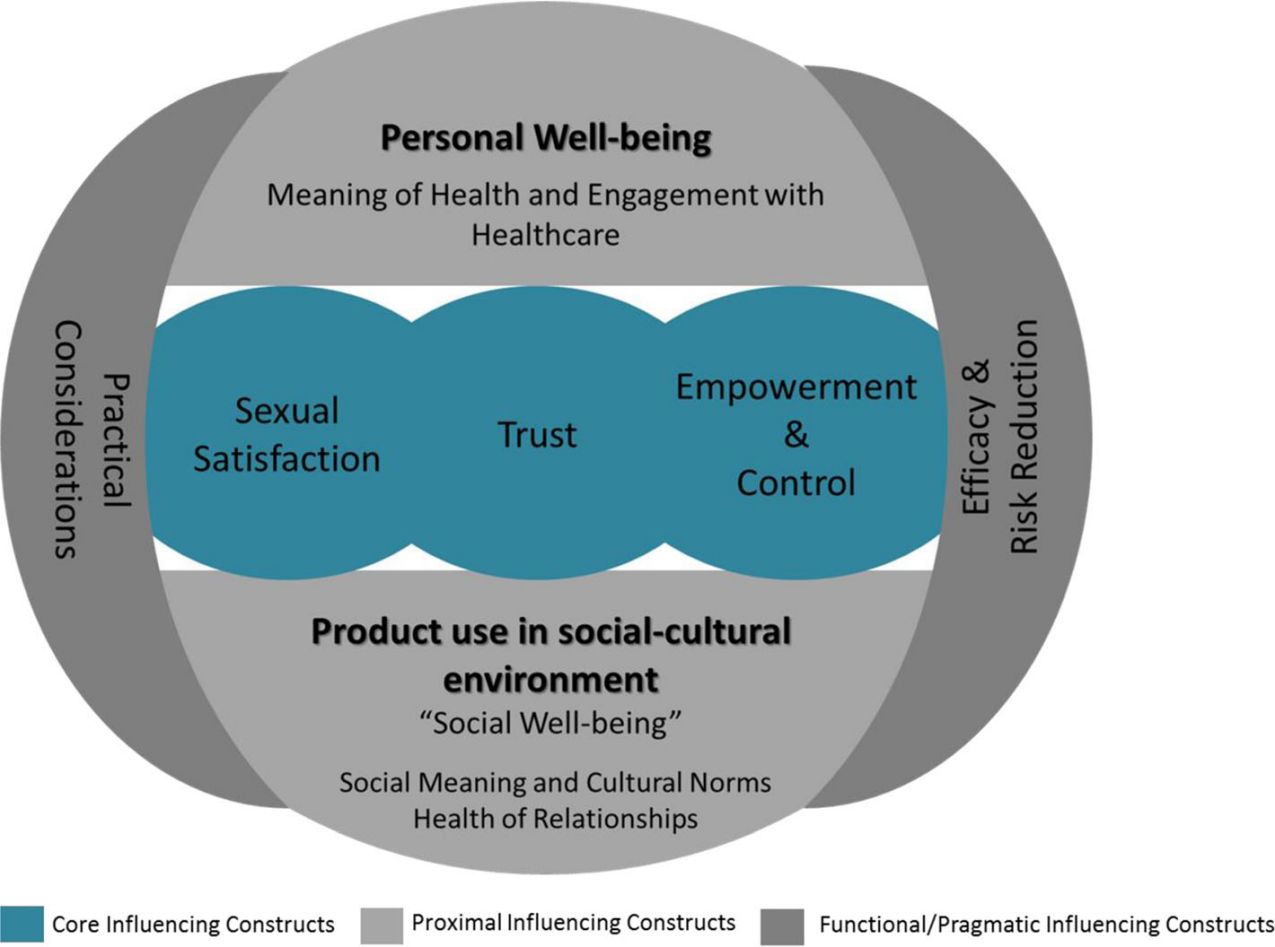
Conceptual framework of third order constructs

We derived 23 s order constructs that we translated into seven overarching third order constructs, mapped in Table 2. The results presented here are organized by third order construct labels. The findings are organised in this way to best illustrate and organise the fluid and inter-relational nature of the themes, which are illustrated in Fig. 3. This is instead of a more binary presentation which has been characteristic of other such reviews [19, 20]. It is also important to note that while this review offers a synthesis of the findings and an overview of the primary themes present in this diverse literature, it was not possible to capture every nuance in all of the selected papers.

**Table 2:** Second and Third order constructs

Third Order Labels	Second Order Constructs	Summary definition (translation) of the 1st and 2nd order constructs	Sources
Sexual Satisfaction	General Sexual Satisfaction	The use of HIV prevention products like the microbicide gel can improve sexual satisfaction within the individual, partner, client, and couple combined.	Gafos et al., 2010; Greene et al., 2010; Montgomery et al., 2010; van der Straten et al., 2012; Okal 2008
	Sexual Performance and Play	Product use can improve performance allowing the user or indiviudal to perform better, be hotter, for her partner, and partners or clients can last longer. There is also the added foreplay of initiating product use (ex. applying the gel).	Guest et al., 2008; Stadler & Saethre, 2011; Montgomery et al., 2010; Stadler et al., 2014; Gafos et al., 2010
	Implications of enhanced satisfaction	Enhanced sexual satisfaction increases trust among some couples, can promote security in the relationship if male partners find their main partners more attractive because of improved sex, and the sense of additional safety from the protection conferred adds to the sexual satisfaction.	Montgomery et al., 2010; van der Straten, et al. 2014
	Lubrication and traditional vaginal practices	Previous intravaginal cleansing and insertion practices can be replaced by product use (ex microbicide), and can improve feeling of sex and feeling of vaginal, making sex more smooth. This more often improves sexual satisfaction, but added wetness can also imply promiscuity in some instances.	Gafos et al., 2010; Greene et al., 2010; Guest 2008; Lees, 2015; Montgomery et al., 2008; Stadler & Saethre, 2011; Montgomery et al., 2010; Sahin-Hodoglugil et al., 2011
Trust	Trust or lack of trust in partner	Product use could be motivated by fear of an unfaithful partner, where they had been and whether they would use a condom. General trust that a partner would use a condom properly was also often missing. In these cases, other HIV prevention products (gel, PrEP, or diaphragm) could confer added protection and peace of mind.	Sahin-Hodoglugil et al., 2011; Kacenek et al., 2012; van der straten et al., 2014; Guest et al., 2008; Kacenek et al., 2010; Sahin-Hodoglugil et al., 2011; Mathenjwa et al., 2012; Lees 1015
	Implications of product use for development and maintenance of trust	Initimacy and creating and maintaining trust are important in relationships where other HIV prevention product use could reaffirm the relationship while condoms carried negative connotations of distrust, denoting infidelity. However, there was sometimes a worry that gels or oral PrEP could promote promiscuity, or at least suggest it.	Okal et al., 2008; van der Straten et al., 2014
	Communication and Enabling Environments	Partner trust of a product was critical, because the trust in the product would translate to trust in a partner as well. Communication and disclosure of product use would improve use of the product, as well as overall communication in the relationship. If not discussed, or if the male partner did not trust the product, there was possibility for arguing and violence.	Montgomery et al., 2008; Stadler & Saethre, 2011; Montgomery et al., 2010, Greene et al., 2010; Montgomery et al., 2012; Montgomery et al., 2014; Montgomery et al., 2008; Kacanek et al., 2012; van der straten et al., 2014; Magazi et al., 2014; Montgomery et al., 2015; Sahin-Hodoglugil et al., 2011; Stadler et al., 2014
Empowerment and Control	Self-esteem and personal agency	Product use had positive affects on personal agency and self-esteem leading women to feel empowered by the ability to decide to use a particular product and that there was something they could use without necessarily needing a male partner’s consent. However, in some cases the product could reduce the sense of personal power if it reminded the user of previous trauma.	Sahin-Hodoglugil et al., 2011; Okal et al., 2008; van der Straten et al., 2012; Mathenjwa et al., 2012; Abrahams et al., 2010; van der Straten 2014; Lees 2015; Stadler & Saethre, 2011; Kacanek et al., 2012; Guest et al., 2008; Greene et al., 2010
	Power positioning (Negotiation and control, Product use and engagement in services affects power dynamic)	Male partners could react negatively to women having decision making power over product use, clinic attendance, or even knowledge that they did not possess. This could result in anger or violence in the household.	Stadler et al., 2014; Montgomery et al., 2015; Montgomery et al., 2012
Personal Well-being	Product use promotes health and well-being	The use of HIV prevention products was seen as a deliberate action to promote one’s own health and sense of well-being. Products could strengthen the sense of self and empowerment, as well as prevent multiple diseases and improve health issues. The physical experience of side effects could also contribute to the sense of protection from the products. The engagement in health services in connection with HIV prevention product use was also a part of seeing onself as being healthy and promoting that image to others.	Stadler & Saethre 2011: Montogomery et al., 2010; Magazi et al., 2014; van der straten et al., 2014
	Quality of care as motivation for engaging in healthcare	The quality of care could motivate or demotivate use of HIV prevention products, negative or positive attitudes from health worker staff would transfer to the individual and promote either their sense of good health or negative feelings towards health.	Van der Straten 2014, Magazi 2014
Social Well-being	Perceived implications of use (how I’m seen by others)	People using products can fear what others will think of them as someone who uses HIV prevention products, largely because of an association with promiscuous sexual activity	Okal et al., 2008; Gafos et al., 2010
	Social construction of medication and product use	The use of a medication can symbolise illness for some women and can challenge their understanding of what it means to be healthy.	van der Straten et al., 2014; van der Straten et al., 2014; Montgomery et al., 2015
	Conflation of ARVs for treatment and prevention	Family members, partners or wider community members can mistake use of ART based PrEP, for ART used to treat HIV infection. This can lead to stigmatisation of people believed to be HIV positive	van der Straten et al., 2014; Magazi et al., 2014; Montgomery et al., 2015
	Interaction with normative vaginal practices and beliefs	The use of vaginal microbicides in some settings compliments locally normative vaginal practices in helping to cleanse the vagina prior to, or after, sex. However, the converse was also observed and vaginal microbicides can be rendered less effectiveness by virtue of cultural norms relating to vaginal cleansing immediately after sex.	Gafos et al., 2014; Greene et al., 2014; Behets et al., 2008, Stadler & Saethre, 2011
	The role of outsiders	Many of the product trials or demonstration projects have been led and/or delivered by people perceived as ‘outsiders’, largely relating to a perception that the originate in the Nothern Hemisphere.	van der Straten, 2014; Guest et al., 2010; Montgomery et al., 2010; Lees, 2015; Montgomery et al., 2014
Practical Considerations	Accessing and storing medication	Physically getting to the clinic to pick up medication or product refills could prove difficult and was an issue in terms of consistent access. Storing medications was sometimes problematic due to stigma within the household or among friends, where personal privacy was minimal.	Greene et al., 2010; Magazi et al., 2014; Montgomery et al., 2010; van der Straten et al., 2014; Abrahams et al., 2010; Mathenjwa et al., 2012
	Taking and adhering to medication	Strategies for using products, such as gel within a certain time period or pills on a daily regimen, could be interrupted by changes in routines or boredom with use. Perceived or actual side effects were also barriers, as was the need to use multiple products such as condoms and gel when wanting to also prevent other STIs or pregnancy. If product use or associated clinic attendance got in the way of livelihood then product use was also demotivated.	Guest et al., 2010, van der Straten et al., 2014; van der Straten et al., 2014; Montgomery et al., 2012,
	Health service level issues	The health service itself, including waiting times at the clinic, required frequency of visits in relation to livelihoods, and transport and ability to get to the clinic could also cause problems in consistent and continued product use.	Magazi et al., 2014
	Product attributes and acceptability	The ease or difficulty in using a product would directly affect whether a product could be taken up and used. These included need for privacy or washing facilities, whether the product stayed where it was supposed to, ability to transport it inconspicuously, and flexibility around when sex occurred. Pain or irritation with use was also a demotivator. Ability to use covertly was positively regarded, even if rarely done.	Okal et al., 2008; Sahin-Hodoglugil et al., 2011; Montgomery et al., 2012; Greene et al., 2010; Kacanek et al., 2012; van der Straten et al., 2014; Guest et al., 2010; Behets et al., 2008; Gafos et al., 2014; Stadler & Saethre 2011; Guest et al., 2008; Mathenjwa et al., 2012; van der Straten et al., 2012
Efficacy and Risk Reduction	Efficacy for HIV prevention central concern	Whether or not the product can effectively protect them from acquiring HIV was a key concern of women engaged with the products via trials or demonstration projects. A recognition that condoms are not always sufficient drives interest in their concern for new product efficacy.	Lees, 2015; Greene et al., 2010; Stadler & Saethre 2011; 2014; van der Straten et al., 2014; Montgomery et al., 2010
	Other (non-HIV) protective effects	While not necessarily acurate in all instances, some female participants expressed beliefs that products could protect them from other STIs or from unwanted pregnancy.	Montgomery et al., 2012; Okal et al., 2008; Mathenjwa et al., 2012; Guest et al., 2008; Behets et al., 2008
	Perceptions around combination prevention	While women may not always be using new technologies in isolation, sometimes a result of concerns for their effectiveness, they were comforted by a feeling that products could provide an additional layer of protection should their primary prevention mechanism (usually condoms) fail.	Sahin-Hodoglugil et al., 2011; Okal, et al., 2008; Guest et al., 2008; Kacenek et al., 2012

### Weight of evidence review

The WoE review found a relatively high level of quality across the body of evidence included in this adapted meta-ethnography. No one category scored lower than medium. Rather we found many medium-high and high ratings. Some papers came from ancillary or imbedded trial research which may not be considered ‘real-world’, and many did not explicitly focus on answering the overall aim articulated in this review. However, these papers were still included because they contained data directly responding to the review aim. All of the studies were conducted with strong, clear methodologies which led us to give the evidence overall a high rating.

## Meta-ethnography

### Sexual satisfaction

Constructs of “Sexual Satisfaction” arose in thirteen of the papers we reviewed [27–39]. These second-order constructs included: 1) general sexual satisfaction; 2) sexual performance and play; 3) implications of enhanced satisfaction; and 4) effects of vaginal lubrication and traditional vaginal practices. Particularly strong positive feelings were voiced in relation to vaginal microbicide gels [27, 28, 32], as a result of the “heat”, or *kusisha* in isiZulu, created through use [27]. This also occurred in relation to diaphragms where many women reported increased vaginal tightness [35], or enhanced stimulation when a partner’s penis made contact with the vaginal ring [33]. At the same time, women also reported negative reactions from some male partners who found the ring to be obstructive during sex [34].

Particularly striking in its primacy was discussion of how product use could form part of sexual performance and play between couples. Several papers describe how the use of microbicide gels and diaphragms was integrated into sexual foreplay, such as the product insertion performed by the male partner [29, 37]. Vaginal microbicide use was also associated with increased libido among some women [32, 37] and viewed as a means of overcoming sexual problems, particularly in limiting premature ejaculation by male partners [32, 36]. A perception that the microbicide gel could lead to tightening of the vagina meant that, as described in two papers, male partners would actively request their female partners to use the product to improve the sexual sensation [27, 37]. In a similar vein, the potential for product use was often seen as facilitating discussion and greater sexual intimacy between partners. Sexual pleasure itself had positive impacts on relationships, improving the performance and play among couples, but also improving the security of the relationship for some women when their husbands stopped seeing other women as a result of improved sexual encounters within the primary relationship [32].

Lubrication played a key role in shaping women’s perceptions of microbicide gel use with the majority of papers reporting a positive impact that helped to make sex feel more smooth or comfortable [27–29, 31, 37, 38]. Inserting the vaginal microbicide gel mirrored the use of other substances inserted into the vagina to create a pleasing environment for both themselves and their male partner [32].

There are others who insert traditional medicines for her to be enjoyable (during sex) . . . I used to love things that are inserted that make you enjoyable. . . . Now that I am old I don’t have that time of going to buy such things. I get help from the gel. [32]

### The multiple dimensions of Trust

We found “Trust” to be a particularly strong, complex, and crosscutting construct, either positively or negatively influencing product uptake and use. From various perspectives, trust was either built up or broken down by interactions with partners in relation to product use. Three second-order constructs emerged under this theme including: 1) trust in one’s partner; 2) implications of product use for development and maintenance of trust; and 3) communication and enabling environments for trust building. These constructs were identified in 16 of the papers [15, 27–38, 40–43].

Women’s lack of trust in their partners was a strong motivator for use of PrEP, female condoms, microbicide gel, and gel with diaphragm [15, 29, 30, 35]. Product use helped ease the fear of possible infections a man might bring home with him, HIV or otherwise, especially when it was difficult to insist upon the use of male condoms within the context of a regular partnership [29, 30, 35].

Product use also had direct implications for the development and/or maintenance of trust within the couple. In several instances, women reported that bringing an HIV prevention product into the home was negatively seen by partners who felt it implied infidelity on their part or could encourage the woman’s promiscuity, thereby impacting their ability to use the products [15, 34].

Conversely, for many couples, the microbicide gel did not convey the same level of mistrust that the condom had, making use easier to negotiate [37, 38]. Communication improved product use, and product use in turn could improve sexual and relationship communication, allowing for new dialogues and trust around sex and intimacy. Disclosure of product use, or lack thereof, also had the potential to influence a woman’s standing in her home and her relationship, where use could result in violence or dissolution of the relationship, or help to improve sexual satisfaction and dynamics within a couple [28, 32, 38, 41, 43].

Partner support of product use was also a critical factor. Some partners plainly refused to use any prevention products citing mood, general disapproval, or dislike of added wetness from microbicide gel use [15, 28, 30]. However, in many instances, men could also be supportive and feel they were protected by the product, as well as become involved in supporting their female partner in use, such as providing transport to clinic appointments [28, 35, 42, 43].

Finally, there was an aspect of trust in the product itself, either negative or positive. Negative perceptions often manifested from male partner’s disapproval and mistrust in outsiders having influence on sexual relationships or in the efficacy of the product. On the other hand, some couples found that a new product with greater efficacy could actually improve trust and feelings of safety that would motivate use, particularly when they had previously found effective condom use problematic.

I like using the diaphragm a lot. My partner likes condoms, but he says they are weak. I also think they are weak [...] [Condoms] burst just like D said. It burst while we were busy [having sex]...So I sometimes use [the condom], but I trust the diaphragm more. [35]

### Empowerment and control

The interrelated constructs of “Empowerment and Control” were central to women’s narratives about how they perceived and used HIV prevention products. Two second-order constructs were identified under this theme: 1) self-esteem and personal agency; and 2) power positioning. These constructs emerged from 14 of the review papers [15, 28–31, 33–37, 40, 41, 44].

Some women expressed how products, in particular microbicide gel, vaginal ring, and diaphragm, gave them a sense of ownership and agency over preventing HIV, but also their own bodies and health [33–35]. They were able to make the decision to use a product, without a man’s consent or involvement. This was especially valuable when women felt that their partners would not necessarily agree or were untrustworthy. Participants suggested that women were responsible for their own health, as this quote notes in relation to the female condom:

Men cannot be trusted to act in our best interests. He can wear the condom at the start of the act and then remove it later or he will just tear it. … So we have to take care of ourselves by using condoms. [40]

In less common contexts, product use can also affect agency, as described in one paper about PEP use within the context of post-rape care. In this paper, successful PEP use after cases of sexual assault was directly related to how the rape was perceived and how the use of PEP affected the victim on an emotional level [44]. Several women reported that the use of PEP reminded them of the rape or made them feel like they were HIV positive, leading to negative associations with the product and demotivated use.

In direct contrast to the generally improved self-agency from product use is the construct of power positioning which emerged as a barrier to product uptake and use. A key concern in this regard was a fear of violence should male partners’ discover covert use of the product.

‘I was scared of the conflict it would cause’; ‘if he finds out he is going to be angry’; ‘I had seen that he didn’t like the gel and I thought if I told him he would fight with me’; ‘I think he will fight with me for using the gel with him in secret...’ (multiple respondents) [36]

Women had conflicting feelings about product use. Some felt product use could improve their ability to make choices and negotiate protection, however, this could also pose a threat to men’s authority and potentially destabilize the relationship [43].

### Personal well-being

“Personal Well-being” arose as an important construct in how women used and engaged with products. We identified three distinct constructs comprising this theme: 1) product use promoted health and well-being; 2) attributes of product use indicated the power of medication and good health; and 3) quality of care was a motivator for engaging in services and product use. These constructs emerged to varying degrees in five of the review papers [15, 32, 37, 39, 42].

Two of the papers [32, 37] explored how a microbicide gel gave women a sense of well-being, solved multiple health issues, and prevented other diseases or infections. Indeed the power of the prevention product was seen to have the ability to promote fertility and vaginal cleanliness, clean the blood, and cure ailments [32].

As a result of continuous use, my pores are now open. My body is no longer stiff and I don’t get tired any more. I am not unsure about my health anymore. Since I started using the gel, I am always energetic like somebody who is using drugs. It has even opened the veins to my kidneys. [37]

Another paper found that the experience of side effects from ARV-based prevention products encouraged perceptions of the power of the ARVs working in the body to protect the user [15, 39].

[T]he tablets are also working because they have some reaction on us like some of us have headaches and become nauseous and stuff like that, so you would believe that means that these tablets have a certain possibility of reducing the risk of contracting HIV, you know. [15]

Interaction with a health service, whether within a trial or actual clinic setting, driven by product use promoted an additional sense of personal well-being in which women could actively look after their own health and be seen by others as ‘healthy’ [15]. Knowledge of one’s HIV status with regular check-ups could promote a negative status, leading to continued product use [42].

Additionally, the quality of care during clinic attendance was directly related to motivation for use in two of the papers [15, 42]. Women noted the importance of staff demonstrating their concern and care for their study participants or clinic clients through educational or one-on-one counselling sessions, in contrast to previous experiences in government public health clinics where staff were often quick to dismiss interests in new products and/or the feelings of their patients [42].

### Product use in the social-cultural environment

The construct of “Product use in the social-cultural environment” incorporates 4 s-order constructs which, combined, represent a significant and sizeable component of the published evidence on the uptake and use of female controlled HIV prevention products [15, 27, 31, 34, 35, 37–39, 42, 43, 45, 46]. The four constructs include: 1) perceived implications of use; 2) dominant, setting-specific social construction of medication and product use; 3) conflation of ARVs for prevention and for treatment; and 4) interaction of products with normative vaginal practices and beliefs. Cutting across these constructs is the notion of how women use products within the social-cultural environment and interactions which point to their need or desire to protect their ‘social well-being’, including their observation of social norms and values.

Many women were concerned that use of vaginal microbicides, the diaphragm, or oral PrEP might suggest to others that they are either promiscuous or identified them as a sex worker [27, 34]. Clinic attendance and use of an ARV based technology also caused confusion for the family and friends of some female participants who struggled to distinguish between ARVs for treatment and for prevention [15, 42, 43]. The use of ARVs has become synonymous with HIV infection and, in some instances, sickness [39] and those using ARV-based prevention products were considered to be ill. As such, the social construction of medication and product use encapsulates particular beliefs regarding use of medications. Therefore, a woman’s own view of how she sees herself, and how she is seen by others, may be threatened by the use of a technology such as PrEP coming in the form of a tablet [42].

Like my family, I explained that I am attending a [PrEP] study but they don’t [believe] that I am attending a study, they just thinking I am HIV positive and I am hiding it. [15]

The impact of beliefs on uptake and use of new products also extend beyond those that relate to HIV stigma. Culturally appropriate or common hygiene practices as they relate to use of prevention products are the focus of several papers within this review [28, 37, 45, 46] and authors highlight the ways in which cleansing practices, in particular, are an important dimension of the social self. Hygiene practices, such as using cleansing products, were reported both as a barrier and an enabler to the effective use and acceptability of vaginal microbicides. Some women found vaginal microbicides highly acceptable given the existing cultural norms around intravaginal insertions for cleansing and preparation for sex [28, 32]. Partner and social preferences for “dry sex” motivated microbicide use which was seen to have cleansing effects on the vagina, translating into a reduction of STIs and foul-odours previously caused by traditional vaginal cleansing products [37]. Interestingly, at least in the South African context, preferences for dry sex seem to actually refer to cleanliness or tightness rather than the desire for a dry vaginal environment, as presented by Stadler and Saethre [37].

The perception and extent of engagement with a biomedical product was also influenced by *who* was delivering the product and whether they were seen as part of the community. Four studies [15, 31, 32, 47] cite concerns relating to the fact that vaginal microbicides, PrEP or the diaphragm were delivered by *muzungu* (white people in Swahili) or people from the northern hemisphere, and a generic mistrust of foreign medications not common in the local setting.

So I sometimes think what if what my friends are saying is true, as they say ‘what if they are infecting you with AIDS using that gel? [15]

In several instances it was male partner mistrust of ‘outsiders’ in their social setting that stood as a significant barrier to uptake and use of the product as they sought to prevent their female partners from engaging in use [43].

### Efficacy and risk reduction

Constructs of perceived and actual efficacy of prevention products, or the potential for risk reduction, were a significant feature of nine papers [28–30, 34, 37, 40, 41, 45] and an implicit dimension of three [31, 32, 39]. Three constructs identified among these papers were 1) efficacy for HIV prevention as a central concern; 2) other (non-HIV) protective effects; and 3) perceptions around combination prevention.

The fear of infection was a dominant feature in participants’ narratives [28, 31] as was the hope that new products may succeed in stemming the epidemic where condoms have been insufficient [37]. In several studies, women said that sex was more enjoyable when they felt protected from HIV, as described in Guest et al.:

It is the diaphragm and gel that made us enjoy sex more because there is no virus that goes inside me or penetrates me. I don't know what he is doing in my absence, and he doesn't know what I am doing in his absence so we are safe when we are using the diaphragm. [29]

Female participants were comforted by the additional protection that new prevention products offered. While they may maintain a desire to utilise male condoms for many sexual encounters (e.g. to prevent pregnancy or other STIs), it was felt that the diaphragm [34, 35] or vaginal microbicides [29] could provide an additional layer of protection from acquiring HIV.

I feel free when the diaphragm inside me in this 6 hours I do simply know that even if it has happened that a condom burst, no HIV will be passed on to me. It will go back. [35]

The preference for use of more than one product at a time affording multiple layers of protection was not uniform. Kacanek et al. (2010) highlight the reluctance of women in their study to use both the diaphragm and condoms simultaneously. However, women did acknowledge that this was partly born from a desire to understand the effectiveness of the diaphragm as a preventative HIV transmission method in isolation.

For female condoms and the diaphragm, women articulated their belief, and feelings of comfort, that these methods could also protect them from other STIs and unwanted pregnancy [29, 34, 40, 41, 45]. The female condom was particularly favoured by women who had experienced problems with hormonal contraceptive methods [40].

### Practical considerations

Four second-order constructs emerged within the third order construct of “Practical Considerations”. These included: 1) accessing and storing products, 2) product attributes and acceptability, 3) ability to effectively take or use the product, and 4) issues relating directly to health services. These constructs were identified in 17 of the papers [15, 28–30, 32–35, 37, 39, 40, 42, 44–47].

While all of the studies included in this review report data on actual use of products in contexts where the products were provided for research purposes, either in trials or clinic settings, issues around access to the products still arose. This was directly related to women’s ability to get to the clinic for refills in between scheduled visits. In some cases women just waited for the their next appointment rather than making an extra trip, or were away from home due to family obligations [28, 42].

Storing the products could also pose problems in settings where there is little privacy in the home and women feared accidental discovery and potentially negative reactions from household members or partners [15, 32]. Some women used the discovery of a product as a means to establish health status and pride around use:

At first I was putting [the tablets] inside my bag and then I took them out of it and put them inside my wardrobe but then one of my friends opened my wardrobe. Because she saw that I was taking the tablets and she didn’t understand why I was taking the tablets even my partner didn’t understand why I was taking the tablets. So I put the tablets in open field so that they could understand that I was taking the tablets for the study and it’s not that I was sick or anything like that. [15]

Attributes of the products themselves could directly influence the ability to use them. With regard to the diaphragm, users found that it could be problematic to insert or remove it without privacy or clean facilities in which to wash themselves and the product. Some women found it painful at first to use, while others appreciated the small size and that it was inconspicuous enough to fit in a handbag or in a pocket [34, 35]. The long-acting attribute of the vaginal ring contributed to a feeling of flexibility when sex occurred, as well as constant protection in case of rape [15].

Vaginal lubrication practices also arose under this third order construct from the practical perspective. After using microbicide gel, women articulated that the use of traditional lubricants was less preferred owing to their ability to cause foul-smelling odours, whereas the microbicide gel or the female condom had built-in, clean lubrication which was a strong motivator for use [28]. This built-in lubrication would also prevent pain and tearing of condoms or vaginal tissues, as well as dryness, which can occur during longer sexual encounters and as such was preferable to male condoms [29, 32, 37]. The vaginal ring could bring added pleasure to sex as well where the ring itself would add stimulation to the male partner [40].

Other papers presented discussions of traditional or conventional vaginal practices and how they might affect the practical and effective use of products [37, 45, 46]. Women spoke of the “dirt” (or pollution resulting from perceived accumulation of semen, menstrual blood, and lubricants), either their partner’s or their own, which could get trapped in their vaginas after sex because of product use [37, 46]. However, the rinsing of a vaginal microbicide gel or diaphragm within an hour after vaginal intercourse could significantly negate its effectiveness. This issue was amplified for female sex workers who felt a need to cleanse their vagina between clients [45, 46]. Interestingly, some women felt that using the newer products actually made them feel cleaner, thus reducing cleansing practices and motivating use.

Side effects, whether real or perceived, were a critical influence on continued use of a product. Women stated they would use products providing there were no visible side effects which could alert friends, family, or partners to their use and potentially stigmatize them as being HIV positive [15]. Women expressed fear of using products due to potential or experienced side effects [15, 33, 39], while others were able to quickly overcome the side effects and felt happy to use the product [28]. Lack of side effects as experienced with the female condom was a big motivator for use, especially for those women who had experienced them with other products.

An additional practical consideration centres on the consciousness required for consistent and correct pill taking, in particular related to oral PrEP. Some women had difficulty remembering to use the product, such as in the case of oral PrEP, when they were intoxicated, “feeling bored or lazy, on the go”, or just not used to having to take a pill every day [15, 28].

Finally, issues with health services, even in trial settings, were also factors influencing product use. Waiting times at the clinic would cause women not to attend and pick up their products, as did availability of and ability to get transport, and family, community, or work obligations which disrupted clinic attendance [42].

## Discussion

The analysis and synthesis of the data included in this review reveal nuanced personal, relational, social and cultural factors that women perceive and attempt to manage as they consider the uptake and use of biomedical HIV prevention products. The factors are far from binary, in that a given factor can act as either a motivator or deterrent to uptake and use depending on the individual context. Our analysis led us to identify seven third-order constructs. Figure 3 illustrates how the third order constructs tend to be situated in terms of primacy to the decision making process to take up and use HIV prevention products, and how they can influence each other in this process.

In this manner, the centrality of “Sexual Satisfaction”, “Trust” and “Empowerment & Control” are emphasised among those constructs that appear more proximal or those that are functional or pragmatic, but which still play a role in product uptake and use. The inter-relationality of these constructs, illustrated by the overlapping circles in the centre of the framework which also connect with the other shapes in Fig. 3, is as important as the individual constructs themselves, as no one construct can be isolated and addressed without acknowledging the others. In the remainder of this section we use this framework as the basis for discussing the broader context of the constructs, their meaning and potential impact for policy and programming.

### Core influencing constructs

Improved sexual satisfaction emerged as one of the strongest motivators for using a particular product, especially in relation to vaginal microbicides and the diaphragm. However, this construct was typically positioned within a partnership where trust and empowerment mediated (or were mediated by) enhanced sexual pleasure. Several authors highlighted how the mere fact that sexual satisfaction for both partners plays such a key role in broader relationship satisfaction, and that in many respects vaginal microbicides or diaphragm use help to enhance sexual pleasure, means that this dimension of the product is central to their uptake and use [29, 32].

Improved sexual satisfaction in a relationship can directly lead to improved security between a couple, where a husband may stop seeing other women and the main partnership therefore becomes strengthened [32]. Product attributes themselves can also contribute to trust, or lack thereof, in a given product. For instance, the added wetness from a microbicide gel could denote infidelity in the eyes of a male partner [28, 36], especially in contexts where traditional preferences derived from social norms are for dryer sex. This connotation of lack of fidelity generates a lack of trust and ultimately demotivated use of the product.

We found that many authors approached their research from the perspective of empowerment, or control over prevention choices. Women across the research expressed how having something that was theirs and/or their choice was important and empowering, but it was not necessarily the primary motivation for use. This is significant to note in the context of a global PrEP and vaginal microbicides discourse that often emphasises empowerment of women as a key component of such products [48–50]. While this review demonstrates that empowerment and control issues still play an important role in the decision to use a new technology, they do not necessarily feature as the primary factor in women’s’ thinking and must be considered within individual contexts.

Empowerment could actually be diminished if a partner discovered covert use of a product and became angry or violent, and fear of these reactions led women either to openly disclose or discuss use with a partner, or stop use altogether. Some women found that empowerment through building trust with their partner and strengthening the relationship through open communication was a positive by-product of introducing new prevention options into the mix.

### Proximal influencing constructs

Product use in the social-cultural environment consists of multiple factors pertaining to interactions between women and their communities, and the social norms generated around traditional practices and beliefs, denoting women’s sense of ‘social well-being’.

Our analysis revealed the importance to women of meeting both cultural norms and expectations of vaginal dryness [31] as well as vaginal cleansing [32]. This is where women carefully consider how vaginal microbicide use might impact on good health and the “vaginal environment” [32], which directly relates to perceptions of well-being in the social-cultural environment as well as sexual satisfaction within the relationship. Several authors suggest that in countries or areas where intravaginal insertion prior to sex (e.g. to tighten or dry the vagina) is commonplace, acceptability of vaginal microbicides – and, therefore, uptake and use – may be higher than in areas where such practices are less common [28, 32].

The construct of personal well-being speaks to the feelings women had about the impact of products on their health more broadly. Use of products meant not only a continuous process of engagement with healthcare services, but also a general sense of health and cleanliness. A further key dimension of this construct was how others might see women as ‘healthy’ by virtue of their on-going healthcare engagement. This observation actually sits in contrast with other findings described in some results that suggest women may be the victims of stigma or discrimination if others come to believe they are accessing HIV services because they are sick, conflating services for prevention with those for (HIV positive) treatment.

### Functional & pragmatic influencing constructs

Inherent to the construct of efficacy and risk reduction is the process of decision-making. Women will engage in balancing the potential risk of HIV with the risk to trust her partner, or the risk to intimacy within a partnership that could be threatened by use of HIV prevention products. Products that pose added benefits from product attributes, such as clean lubrication from the female condom or microbicide gel, could outweigh potential issues around distrust or scepticism around product use.

Several authors stressed that the efficacy of the technology in preventing the transmission of HIV was absolutely central to its perceived acceptability [28, 29, 32], as well as its uptake and use. However, we found that sometimes beliefs about a certain product was as, or more powerful than the reality. For example, the belief that a product could prevent more than HIV, that it could ‘cleanse the blood’ or prevent other diseases because of how it made the person feel when using or taking it, was a strong motivation for use. Similarly, belief that a product was extremely efficacious as a result of experiencing side effects or a feeling of empowerment promoted continued use.

In order to make effective use of the products women need to be able to store, be able to confidently use, and have barrier-free access to healthcare services. Products must also be developed or formulated in such a way as to be acceptable to women wanting to use them in the medium or longer term. Vaginal microbicides and the diaphragm, in particular, can be difficult for women to insert in adequate time prior to sex and concern was also expressed that their use may interfere with traditional vaginal hygiene practices.

### Further reflections on these findings

Since the time that this review was conducted, additional qualitative evidence on perceptions of female-initiated and controlled HIV prevention products or interventions has emerged, particularly from trial research around oral PrEP.. The HPTN/ADAPT study [51], which compared daily to intermittent dosing of oral PrEP in a phase II clinical trial setting, has recently published qualitative research which further supports and develops the findings in this paper. In this study, they found nuanced motivations and barriers linked to perceptions of safety taking PrEP, trust in what PrEP really was and whether it really worked, whether its providers were worthy of trust, and a sense of community commitment and dedication with regards to adopting (or not) the PrEP intervention. The insights related to trust, or lack thereof, in oral PrEP have been further supported by findings from the VOICE D study (a sub-study of the main VOICE study on preferences and adherence) where women expressed concerns around safety and efficacy of PrEP as well as similar community perceptions which affected their own thinking [52, 53]. As more data continue to emerge from ongoing trial research as well as the early PrEP implementation studies which will start to publish results, perceptions which might create product-specific stigma, distrust, and aversion will be important to consider when designing messaging and education campaigns. Additionally, it will be important to ‘arm’ health workers and clinicians providing PrEP will accurate information and careful training to be able to address rumours head on.

In this review, oral PrEP was discussed less than other products (such as microbicide gels or the ring) with regard to sexual satisfaction, largely because it lacked tangible attributes that could contribute to improved pleasure. However, social marketing campaigns have evolved since this review was conducted that capitalize on the ability of PrEP to remove some of the fear around sex which can then lead to improve satisfaction or pleasure [54]. PrEP and other HIV prevention products may present a new opportunity to develop discourse around sexual pleasure even in places traditionally closed to these notions.

Vaginal practices played a key role in much of the research surrounding vaginal products such as the gel and the ring with regards to traditions and habits of women. Interestingly, the notion of “dry sex” was revealed in some of the literature to be less about having a dry vaginal environment, but rather more about the cleanliness of the vagina and “hotness” of the sex. Regular clinic visits allowed women to be more consistently free of STIs, and use of the gel provided clean lubrication, which most women and their male partners enjoyed. If there is to be continued pursuit of vaginal HIV prevention products, this line of enquiry may benefit from further investigation.

Adherence, or the burden of adhering to a specific HIV prevention product regimen, was not a theme that emerged as prominently as others in this review. However more evidence has emerged recently about levels of adherence required to ensure efficacy, and findings from qualitative research may help to shed light on where issues lie in maintaining consistent use. Oral PrEP in particular requires high levels of adherence to confer adequate protection, particularly among women [55, 56]. This will also be an important consideration for large-scale PrEP implementation as well as for development of new HIV prevention products.

Finally, as the research included in this review comes from studies where participants received some form of financial reimbursement for participation, and the products were provided free of charge, it is not possible to determine how perceptions of use may differ in a context where this was not the case. Future research could examine this further, and potentially use the findings from this paper to develop surveys to also quantitatively assess motivations and barriers to use in more ‘real-world’ settings.

### Strengths & Limitations

This is the first review paper of its kind to aggregate data from across a large population of women, from multiple sub-Saharan African countries, relating to a range of female controlled HIV prevention products. In doing so we have documented the key themes or issues that can influence their uptake and use and how these overlap according to context and the specific technology. The systematic review involved searches in all languages, double screening and double extraction of relevant data from each paper. Our explicit focus on studies where the product of interest was available, rather than simply the object of theoretical discussion with women, helps to ensure the findings are grounded in personal experience.

The papers in this review, however, included mostly data gathered in the context of clinical trials where products were freely given and participants were usually reimbursed for costs associated with participation (such as transport to and from clinics). The unique environment and provision of wrap-around counselling and treatment services does not often mirror the real-world implementation of new products. Nevertheless, regardless of this, the themes that emerged were common across a wide range of countries, implementation contexts (including clinical trial and implementation studies), and cultural settings, as well as across the products themselves, suggesting a relatively robust body of evidence. One crucial consideration, however, relates to the construct of product use in the social cultural-environment, where social norms are seen to influence uptake and use of products. As those products found to be efficacious are rolled out into countries and communities, the extent to which their use is ‘normative’ will also change. This would certainly have affected the data included in this review as none of the products had been in the field long enough for use to have been normalized. It is also important to note that while this review of evidence only included perspectives from women, it is clear that the perspectives of men influence women’s choices, decision making, and effective use.

A further limitation of this analysis is the limited nature of published qualitative research. Often it is required, for word length purposes, to condense detailed findings to a few sentences that capture their essence. Understandably, the papers included in this review will have focused on certain viewpoints, whereas there may be many more data providing additional nuance, which could contribute valuable insight.

Finally, while it is a strength of this review that we have synthesized qualitative data from across research contexts, it is also somewhat of a limitation that the contexts were not more diverse and include more pure implementation research studies. The body of evidence for understanding the motivations and barriers to uptake and use of female-initiated HIV prevention products can be further strengthened by more lessons learned and documented from the field.

## Conclusions

This is an exciting time in HIV prevention as new biomedical products are developed and systems put in place to ensure their effective rollout. Health systems, structural and direct contact level interventions relating to new products need to take into account more than just superficial notions of acceptability. Instead, they should focus on a holistic approach including: aspects of how a product can protect a partnership (in terms of physical and emotional health); an awareness of the significance of sexual satisfaction and enjoyment; an understanding of the social and cultural norms influencing product use; and include efforts to tackle the continuing stigma associated with HIV. Structural and individual level interventions aiming to improve uptake and use of those products already available have tended to focus on practical issues (e.g. access to products and services, ability to use and store products safely, access to hygienic facilities). These may be perceived as the ‘low hanging fruit’ as issues that lend themselves to immediate intervention, but they do not necessarily reflect those concerns that are most central to effective uptake and sustained use of the product from the perspective of the user. While the reduction of transmission risk is essential for the use of any of these products, and has justifiably been the focus of international scientific attention, this is not often the key determining feature of use. Formative research conducted in each setting prior to the roll out of new product-based interventions will help to ensure that communications and educational materials are aligned to the local cultural and social norms, and take account of both personal concerns and personal values in safer, and enjoyable, sexual practice.

## Abbreviations

ART: Antiretroviral Treatment
ARV: Antiretroviral
CRD: Centre for Reviews and Dissemination
HIV: Human Immunodeficiency Virus
PEP: Post-exposure prophylaxis
PrEP: Pre-exposure prophylaxis
PRISMA: Preferred Reporting Items for Systematic Reviews and Meta-Analyses
STRIVE: Structural Drivers of HIV
WHO: World Health Organization

## Footnotes

1 Microbicide gel can also be referred to in the literature as topical PrEP, however, in this review we have kept is as microbicide gel, or just gel.
